# Uptake of *Plasmodium falciparum* Gametocytes During Mosquito Bloodmeal by Direct and Membrane Feeding

**DOI:** 10.3389/fmicb.2020.00246

**Published:** 2020-03-03

**Authors:** Arthur M. Talman, Dinkorma T. D. Ouologuem, Katie Love, Virginia M. Howick, Charles Mulamba, Aboubecrin Haidara, Niawanlou Dara, Daman Sylla, Adama Sacko, Mamadou M. Coulibaly, Francois Dao, Cheick P. O. Sangare, Abdoulaye Djimde, Mara K. N. Lawniczak

**Affiliations:** ^1^Wellcome Sanger Institute, Hinxton, United Kingdom; ^2^MIVEGEC, IRD, CNRS, University of Montpellier, Montpellier, France; ^3^Malaria Research and Training Centre, University of Science, Techniques and Technologies of Bamako, Bamako, Mali

**Keywords:** malaria, transmission, gametocyte, mosquito feeding, *Plasmodium falciparum*

## Abstract

*Plasmodium falciparum* remains one of the leading causes of child mortality, and nearly half of the world’s population is at risk of contracting malaria. While pathogenesis results from replication of asexual forms in human red blood cells, it is the sexually differentiated forms, gametocytes, which are responsible for the spread of the disease. For transmission to succeed, both mature male and female gametocytes must be taken up by a female *Anopheles* mosquito during its blood meal for subsequent differentiation into gametes and mating inside the mosquito gut. Observed circulating numbers of gametocytes in the human host are often surprisingly low. A pre-fertilization behavior, such as skin sequestration, has been hypothesized to explain the efficiency of human-to-mosquito transmission but has not been sufficiently tested due to a lack of appropriate tools. In this study, we describe the optimization of a qPCR tool that enables the relative quantification of gametocytes within very small input samples. Such a tool allows for the quantification of gametocytes in different compartments of the host and the vector that could potentially unravel mechanisms that enable highly efficient malaria transmission. We demonstrate the use of our gametocyte quantification method in mosquito blood meals from both direct skin feeding on *Plasmodium* gametocyte carriers and standard membrane feeding assay. Relative gametocyte abundance was not different between mosquitoes fed through a membrane or directly on the skin suggesting that there is no systematic enrichment of gametocytes picked up in the skin.

## Introduction

Transmission of *Plasmodium falciparum* from humans to mosquitoes depends on the sexual phase of the parasite’s life cycle. Both male and female gametocytes have to be picked up in the mosquito blood meal in order to mate and colonize the mosquito. Relatively low circulating gametocyte densities are typically observed (Bousema and Drakeley, 2011), and yet transmission remains efficient even at gametocyte densities for which random sampling of the host cannot explain successful infection of the mosquito (Lawniczak and Eckhoff, 2016). Sub-patently infected carriers that harbor parasites below the limit of detection by microscopy can readily infect mosquitoes (Schneider et al., 2007; Ouédraogo et al., 2009). Mosquitoes fed directly on the skin have also been found to be infected more readily than by a direct membrane feeding assay (Bonnet et al., 2000; Diallo et al., 2008). These findings have led to the hypothesis of gametocyte biology and behavior that enhance their transmission but have yet to be described (Pichon et al., 2000; Bousema and Drakeley, 2011). A pre-fertilization behavior, whereby malaria gametocytes associate in the circulating blood and/or adhere to sub-dermal capillaries could enhance their probability of being ingested in sufficient quantities. Intriguingly, two early studies revealed that gametocytes were on average three times more concentrated in skin biopsies than in the venous circulation (Chardome and Janssen, 1952; Van Den Berghe et al., 1954), although these experiments lacked appropriate controls. Whilst associative behaviors have been hypothesized, they have never been explicitly tested. Comparing gametocyte uptake in skin-fed and membrane-fed mosquitoes could determine if the patterns differ between these blood sources. Such an undertaking, however, requires a sensitive detection tool able to accurately measure gametocyte densities in individual mosquito blood meals. The most commonly used gametocyte molecular detection tools, albeit of good sensitivity, only detect female gametocytes in human blood sample (Schneider et al., 2015). In this study, we describe a molecular assay capable of measuring relative gametocyte densities directly in mosquito bloodmeals and investigate the uptake of gametocytes during natural and artificial blood feeding of symptomatic malaria patients.

## Materials and Methods

### Gametocyte Specific Transcript Selection for Ultrasensitive Detection and Multiplex Rt qPcr

In order to find a suitable gametocyte biomarker, 107 transcripts displaying a 25-fold enrichment in gametocytes compared to rings in a published stage-specific RNAseq study were selected (López-Barragán et al., 2011; Supplementary Table S1). qPCR primers to each of these transcripts were designed using the IDT PrimerQuest^®^ Tool. *P. falciparum* isolate NF54 was maintained in O+ blood in RPMI 1640 culture medium (GIBCO) supplemented with 25 mM HEPES (SIGMA), 10 mM D-Glucose (SIGMA), 50 mg/L hypoxanthine (SIGMA), and 10% human serum in a gas mix containing 5% O_2_, 5% CO_2_, and 90% N_2_. Human O+ erythrocytes were obtained from NHS Blood and Transplant, Cambridge, United Kingdom. None of the blood products used contained identifying information from donors. *Plasmodium* culture using human serum and erythrocytes from donors has been approved by the NHS Cambridgeshire 4 Research Ethics Committee (REC reference 15/EE/0253) and the Wellcome Sanger Institute Human Materials and Data Management Committee. Pure ring stage parasites (10^9^) were produced by double Percoll-sorbitol synchronization (Moll et al., 2008), followed by negative selection on a MACs LS column (Miltenyi Biotec). Pure stage V gametocyte samples (10^8^) were produced and purified on a MACs LS column (Ribaut et al., 2008). Parasite counts were established by Giemsa staining and hematocrit was measured with a hemocytometer. RNA was extracted with TRIzol according to the manufacturer’s recommendations. RNAs were treated with DNA-free DNAse Turbo kit (Ambion) and reverse transcribed with the high capacity reverse transcriptase kit (Thermo Fisher), supplemented with oligo-dts (Thermo Fisher) at a final concentration of 2.5 μM. SYBR-green qPCR (Roche) was conducted on a Lightcycler 480 (Roche) for each of these transcripts on pure gametocyte and ring duplicate cDNAs. The DNA-free DNAse Turbo kit removes gDNA, a reverse transcriptase-less control was run with each extraction batch and was verified to be negative before inclusion of samples in the dataset. Enrichment of transcripts in gametocytes was estimated using the delta-delta *Ct* method for each 107 transcript and the housekeeping gene glucose-6-phosphate dehydrogenase-6-phosphogluconolactonase (PF3D7_1453800) as a control. A probe-based multiplex assay was designed to allow relative quantification and comparison of gametocytes within mosquito bloodmeals. A triplex qPCR assay was devised: it detected the gametocyte biomarker (*MDV-1*, PF3D7_1216500, cy5), an asexual transcript (*Mahrp2*, PF3D7_1353200, HEX) and a human transcript (*hsGAPDH*, FAM) (Supplementary Table S2). The *Mahrp2* transcript was selected because it was the most enriched transcript in rings compared to gametocytes by RNAseq; if one excludes *Hrp2* or *Hrp3*, two gene*s* that have been shown to be deleted in some populations of parasites and are therefore not suitable (Watson et al., 2017). An alternative assay using the human transcript *UBA-1* was included to validate *hsGAPDH* as a suitable loading control for leukocytes. Multiplex primer efficiencies were calculated on qPCRs of gDNA serial dilutions for all primer/probe combinations (Supplementary Table S3). The qPCR conditions were as follows, the samples were first incubated for 10 min at 95°C, then 45 cycles were performed (95°C for 10 s, 60°C for 10 s, and 68°C for 20 s), followed by a melting curve step to ensure single product amplification. Single amplification was observed for all samples. The melting temperature were chosen as that recommended by the assay manufacturer without alteration. The *Ct* calculated by the relative quantification protocol of the Light cycle 480 software were used. Fold changes were calculated by the delta-delta *Ct* method corrected for primer efficiency (Pfaffl, 2004; p. 96 equation 3.5).

### Mock Blood Meals With a Gametocyte Serial Dilution

To test the triplex qPCR assay with differing concentrations of gametocytes, *in vitro* cultured asexual and sexual parasites were artificially fed to mosquitoes. A serial dilution of gametocytes (10–100,000 per blood meal) was spiked with 10,000 rings per μl, placed in whole blood and spun at 800 *g* for 5 min, heat-inactivated serum was used to resuspend the pellet to 50% hematocrit and aliquoted into a heated plastic membrane feeder covered with stretched parafilm (Bemis NA). Eight female *Anopheles coluzzii* (N’Gousso strain) were allowed to feed on each dilution for 12 min and were immediately sacrificed in 70% ethanol. Fed mosquitoes were tapped briefly on absorbent paper to remove excess ethanol and immediately immersed in 50 μl of TriZol in RNAse-free 1.5 ml tubes and stored at −80°C. Upon thawing, 450 μl of fresh TriZol was added to each 50 μl sample, and mosquitoes were homogenized with a clean pestle and RNA extraction and cDNA generation were conducted as described above.

### Study Site and Ethical Approval

To test the assay in natural conditions, we recruited *P. falciparum* positive patients in Faladie, Mali. Faladie is situated in the Koulikoro region of Mali and is characterized by a seasonal hyperendemic transmission of mostly *P. falciparum* malaria. Patients, aged 6–14 years, with symptomatic non-severe malaria were recruited from November to December 2016 (Table 1). Patients were screened by thick smear microscopy during which the asexual parasite (rings) and gametocyte counts (stage V) were recorded for 1000 leukocytes. A standard concentration of 8000 leukocytes per μl was used to calculate parasitemia and gametocytaemias. The protocol was approved by an IRB from the Faculty Of Medicine, Pharmacy and Odontostomatology de Pharmacy; Université des Sciences, Techniques et Technologies of Bamako (IRB approval letter no. 2016/133/CE/FMPOS).

**TABLE 1:** Feed data for six patients. Parasitemia and gametocytemia were established by microscopy.

**Patient number**	**Parasitemia-day 0 (Microscopy) (/μl)**	**Gametocytemia day 0 (Microscopy) (/μl)**	**Membrane feeder 1 (positive blood meals/total)**	**Membrane feeder 2 (positive blood meals/total)**	**Skin feed left (positive blood meals/total)**	**Skin feed right (positive blood meals/total)**	**Variance (*F*-test)**		***t*-test**	
							**Variance in skinfed than membrane fed**	***p*-value**	**Mean fold change in skinfed vs membrane fed**	***p*-value**
EGF006	14,680	520	16/17	15/17	16/17	12/13	Greater	0.03091	NS	0.1986
EGF007	37,640	48	17/18	6/8	19/20	14/15	Greater	0.006914	NS	0.05617
EGF009	4800	144	15/15	11/12	17/19	10/10	NS	0.1377	NS	0.617
EGF014	1440	360	16/17	9/9	15/15	19/20	Greater	0.01363	NS	0.2308
EFG017	512	296	21/21	20/21	23/23	23/23	Less	0.01764	NS	0.3374
EFG020	17,520	96	14/21	12/17	9/21	11/24	Less	2.545E-06	less	3.06E-05
**Overall**	NA	NA	NA	NA	NA	NA	Less	8.138e-06	NS	0.05251

*Number of fed mosquitoes with a positive *MDV-1 Ct* (<38) is given together with the total number of mosquitoes for each feed.*

### Mosquito Feeding Assay

To compare the number of gametocytes acquired in the mosquito bloodmeal from feeding on the skin or peripheral bloodstream, a peripheral blood sample was first obtained from *P. falciparum* gametocyte carriers in a vacutainer with EDTA and immediately placed into two plastic membrane feeders covered with parafilm (600 μl each). 50 starved *A. coluzzii* females were allowed to feed on each feeder (*n* = 100 total). Membrane feedings were allowed to proceed for 12 min. Concomitantly 2 pots of 25 mosquitoes were allowed to direct skin-feed on each volunteer (back of left calf, right calf) until repletion (8 min approximatively). Following the feeds, all mosquitoes were immediately sacrificed with 70% ethanol. Mosquitoes were each transferred to 50 μl of TriZOl and kept in liquid nitrogen or at −80°C until extraction.

### Mosquito Sample Processing for Parasite Quantification

For RNA extraction, upon thawing, 450 μl of fresh TriZol was added to each 50 μl sample, and mosquitoes were homogenized with a clean pestle. RNA extraction and cDNA generation were conducted as above. Two assays per sample were run: the triplex mentioned above (*MDV-1*, *hsGAPDH*, and *Mahrp2)* and a duplex with the alternative human transcript *hsUBA-1* (FAM) and a control mosquito transcript (ribosomal protein S7, HEX). Samples that yielded *Ct* > 38 for *MDV-1* or *hsGAPDH* were discarded. Fold changes were calculated using the delta-delta *Ct* method corrected for primer efficiency (Pfaffl, 2004; Supplementary Table S4); the test gene was always *MDV-1* whilst the control genes used were either *hsUBA-1*, *Mahrp2* or *hsGAPDH*.

### Sampling and Statistical Evaluation

Statistical analyses were conducted in R and using package “car” for the ANOVA (Fox and Weisberg, 2018). *F*-tests were run with the var.test() function.

## Results

### An Assay to Measure Gametocyte Uptake in the Bloodmeal

**FIGURE 1:**
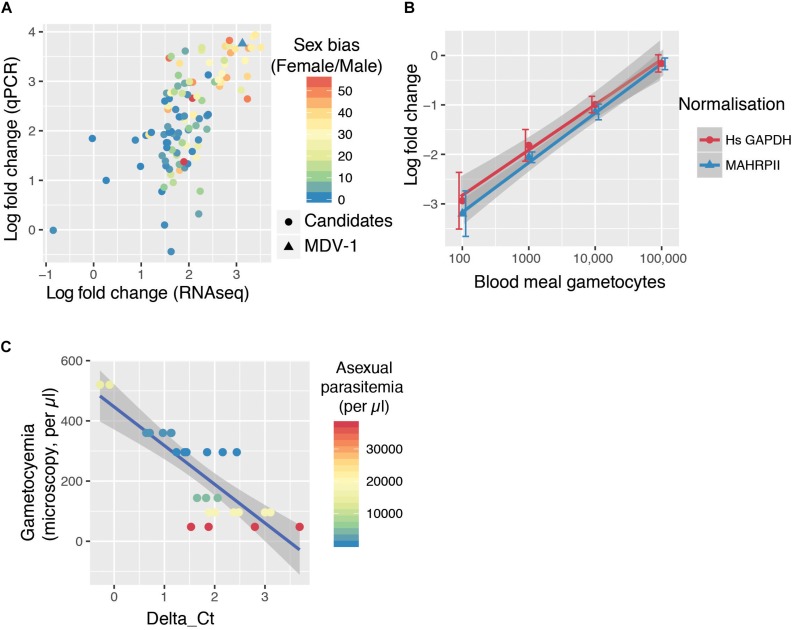
The GQA, a multiplex assay capable of sensitively detecting gametocyte uptake by mosquitoes. **(A)** RNAseq (López-Barragán et al., 2011) and qPCR fold change of 107 transcripts in gametocytes compared to ring stage parasites. The color scale indicates sex-bias in expression based on (Lasonder et al., 2016) with high female:male ratios in red and balanced ratios in blue. **(B)** Relative abundance of MDV-1 in blood meals of mosquitoes fed on a serial dilution of gametocytes compared to the maximum gametocyte input (100,000/bloodmeal). Error bars represent the standard deviation. **(C)** Relationship between observed gametocyte numbers by microscopy and delta *Ct* of test and control gene in 10 μl blood samples from symptomatic malaria patients colored by asexual parasitemia (26 samples from 6 patients).

Because a mosquito bloodmeal is composed of only a few microliters of blood, we reasoned that maximum sensitivity in detecting bloodmeal gametocyte density will be achieved by detecting the most abundant transcripts found in gametocytes. We selected 107 transcripts that were at least 25-fold enriched in gametocytes over the ring form from a previous bulk RNAseq study (López-Barragán et al., 2011). We designed individual RT-qPCR for the candidate transcripts in order to confirm their relative abundance. Previous bulk RNAseq work and our specific qPCR results on 107 transcripts were in good agreement (Figure 1A). We also overlaid a sex-specific transcriptome analysis (Lasonder et al., 2016), which revealed that the most abundant transcripts tend to be contributed by female gametocytes (Figure 1A). The most abundant transcript by both qPCR and RNAseq that did not show a sex bias was *MDV-1* (sex-bias = 1.01).

We tested the sensitivity of this potential gametocyte biomarker by qRT-PCR with a serial dilution of *in vitro* cultured gametocytes mixed with purified ring stage parasites in whole blood (spanning the range of 10–100 k per bloodmeal with a blood meal considered to be 2 μl). *A. coluzzii* females were fed on this serial dilution and immediately sacrificed, and whole mosquitoes were used to generate cDNA. *MDV-1* transcript abundance was normalized with either *Mahrp2*, which is a ring stage transcript, or with *hsGAPDH*, which is transcript present in human leukocyte. The gametocyte numbers estimated to be present in each dilution was significantly correlated to the fold changes normalized with *Mahrp2* (*r* = 0.79, *p*-values < 0.001 by Pearson’s correlation) and *hsGAPDH* (*r* = 0.76, *p*-values < 0.001 by Pearson’s correlation), indicating that the gametocytemia can be relatively quantified by these assays within 8 individual mosquito bloodmeals per condition over a wide dynamic range (10–100,000 gametocytes per blood meal) (Figure 1B). We found only 3 of 8 mosquitoes fed on a blood meal with 10 gametocytes had a signal below the limit of detection threshold (*Ct* < 38), suggesting the limit of reliable detection for the assay is between 10 and 100 gametocytes per bloodmeal. We named this assay gametocyte quantification assay (GQA).

We next tested the GQA on mock blood meals composed of 10 μl of venous blood from 6 symptomatic gametocyte carriers recruited in Faladje and one unfed mosquito (Table 1). We observed that peripheral gametocytemia as measured by microscopy is highly correlated with the delta Ct from the GQA (*r* = −0.82, *p* = 3.567e-07 by Pearson’s correlation) and was independent of peripheral asexual parasitemia (Figure 1C). No single nucleotide polymorphism with a minor allele frequency superior to 0.002 was found in the MDV-1 region targeted by the GQA > 3000 genomes (MalariaGEN Plasmodium falciparum Community Project, 2016), indicating that genetic diversity in natural populations is unlikely to affect the performance of the GQA. Altogether these results indicate that the GQA can be used to measure relative gametocytaemias in minute quantities of blood in both cultured parasites and for wild populations of parasites, it should be noted that the assay will be most useful for densities of gametocytes detectable by microscopy and not sensitive enough for subpatent gametocyte carriage.

### Uptake of Gametocytes During Natural and Artificial Bloodmeals

**FIGURE 2:**
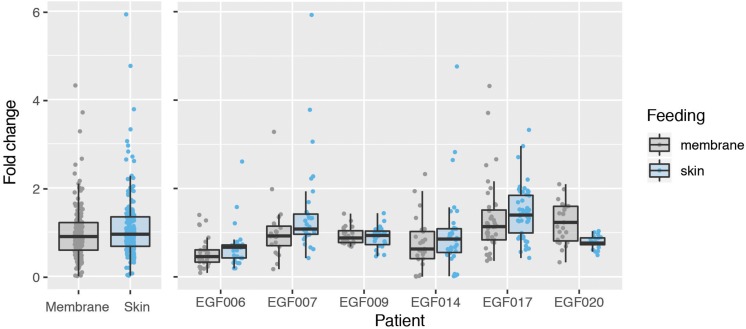
Comparison of gametocyte density in natural and artificial mosquito blood meals. Gametocyte detected in mosquitoes fed on six patients either by direct skin feeding or membrane feeding. The left panel shows the data pooled across patients and the right displays results for each individual. Hinges correspond to the first and third quartiles, whiskers extend 1.5 interquartile range on either side of the hinges. Jitter points are overlaid, those beyond the whiskers are outlying points.

We next used the GQA to measure the relative uptake of gametocytes during mosquito blood feeding either directly from the skin or through an artificial membrane to test the potential role of the skin in gametocyte transmission biology. For the aforementioned six patients, we performed the GQA measurement after direct or membrane feeding. Control samples used in the quantification for each volunteer were mock blood meal samples (see above). We used the linear model: *fold change* ∼ *feed* + *patient* + *feed* × *patient* (where *feed* is the feeding mode, skin or membrane). Relative gametocyte abundance was not different between mosquitoes fed through a membrane or directly on the skin when normalized to human *GAPDH* (type III ANOVA, *p* = 0.39331) (Figure 2 and Supplementary Table S4), an alternative human transcript *UBA-1* (type III ANOVA, *p* = 0.18121), or the asexual transcript *Mahrp2* (type III ANOVA, *p* = 0.6999) (Table 2). This is indicative that there was no difference in density of gametocytes during natural or artificial blood meals. We did, however, note a patient-dependent effect on feeding mode but with inconsistent directionality (type III ANOVA, feed × patient: *p* = 0.01744). We, therefore, tested differences in the fold change of the gametocyte marker for each patient and found no difference in skin-fed vs membrane-fed except in the case of one patient (EGF020, Table 1). We also note that the variance observed between skin-fed and membrane-fed in each patient were different in 5 of the 6 patients but again with inconsistent directionality (Table 1).

**TABLE 2:** ANOVA full results.

**Reference gene**	**Model component**	***F*-value**	**Pr(>*F*)**
*GAPDH*	Feed	0.7305	0.39331
*GAPDH*	Patient	6.6312	6.461e-06
*GAPDH*	Feed × patient	2.7878	0.01744
*UBA-1*	Feed	1.7948	0.18121
*UBA-1*	Patient	5.5182	6.612e-05
*UBA-1*	Feed × patient	2.6143	0.02445
*Mahrp2*	Feed	0.1488	0.6999
*Mahrp2*	Patient	1.7555	0.1214
*Mahrp2*	Feed × patient	1.4370	0.2102

## Discussion

Malaria transmission from human to mosquito is a very efficient process that is still not completely understood (Bousema and Drakeley, 2011). In this study, we have established a new tool to study *P. falciparum* transmission in natural infections. The GQA detects a new biomarker of *P. falciparum* gametocytes (*MDV-1*) which is both sensitive and detects both males and female gametocytes. The GQA is particularly apt at comparing blood samples originating from the same individual because gametocyte densities can be normalized to either asexual parasite or leukocyte transcripts, which should be good loading controls within an infection. The GQA will, therefore, be extremely useful to assess the spatial and temporal heterogeneity in gametocyte densities within an infection. Moreover, this assay requires very little initial input, therefore it can be used for finger prick blood samples, skin punctures or mosquito blood meals, as we have shown in this study. It should be noted that the GQA is used at relatively high gametocytaemias (>100 gametocyte/μl) compared to lower gametocytaemias that may still yield successful mosquito infections.

Associative behaviors of gametocyte pre-fertilization have been postulated as a mechanism for enhanced transmission (Pichon et al., 2000; Lawniczak and Eckhoff, 2016; Nixon, 2016). In this study we have not seen a different density of gametocytes in mosquito bloodmeals taken through skin feeding versus membrane feeding on volunteers with patent gametocytemia. Therefore, in our gametocyte concentration window of observation (48–520 gametocytes/μl) there may not be a differential uptake of gametocytes during skin feeding. Another recent study comparing capillary and venous gametocyte densities also found no difference (Sandeu et al., 2017). Gametocyte clustering in the skin has previously been hypothesized to potentially enhance transmission (Pichon et al., 2000; Bousema and Drakeley, 2011; Lawniczak and Eckhoff, 2016). The effect of feeder type on the variance observed between mosquitoes was also patient dependent. Therefore, if clustering mechanisms do occur, they may not be specifically associated with the skin or may be only observed post-fertilization. There does not appear to be an association between higher variance in skin feeding vs membrane feeding and parasitemia or gametocytemia, noting that our study only examined six patients. Our results warrant further examination of these questions over a larger sample size both in terms of patients and fed mosquitoes and also on volunteers with a wider range of gametocytemia, including those without light microscopy detectable gametocytemia. Indeed, pre-fertilization behaviors might only be apparent at lower gametocyte concentration when the likelihood of infection is low. Additionally, associative behaviors between the sexes that ensure the presence of a male and a female in a blood meal but don’t alter local gametocyte densities, for instance syzygy as observed in a species of *Leucocytozoon* might also be occurring (Barraclough et al., 2008).

Overall the skin has been implicated as an organ that can enhance vector-borne pathogens, including malaria transmission to (Chardome and Janssen, 1952; Van Den Berghe et al., 1954) and from the mosquito (Gueirard et al., 2010) as well as in trypanosomes (Capewell et al., 2016). Conducting a full-scale investigation of the role of this organ in pathogen transmission is an important endeavor and will be facilitated by sensitive tools such as the GQA and may allow a deeper understanding of the infectious reservoir of malaria and how to eliminate it.

## Data Availability Statement

All datasets generated for this study are included in the article/Supplementary Material.

## Ethics Statement

The studies involving human participants were reviewed and approved by the Faculty of Medicine, Pharmacy and Odontostomatology de Pharmacy; Université des Sciences, Techniques et Technologies of Bamako (IRB approval letter No. 2016/133/CE/FMPOS). Written informed consent to participate in this study was provided by the participants’ legal guardian/next of kin.

## Author Contributions

AT, DO, AD, and ML designed the study. AT, DO, CM, AH, ND, DS, AS, MC, FD, and CS conducted the field work. AT and KL performed the molecular biology. VH provided statistical expertise. AT, VH, and ML wrote the manuscript with contributions from other authors. All authors read and approved the manuscript.

## Conflict of Interest

The authors declare that the research was conducted in the absence of any commercial or financial relationships that could be construed as a potential conflict of interest.

## Supplementary Material

TABLE S1 List of genes tested as gametocyte biomarkers.

TABLE S2 Primer sequences in the multiplex assay.

TABLE S3 Primer efficiencies.

TABLE S4 Full qPCR data of skin and membrane feeds.
